# Cross-Disciplinary Mentoring and Collaboration to Advance Preventive Gerontology

**DOI:** 10.1093/geroni/igaf122.957

**Published:** 2025-12-31

**Authors:** Jeff Williamson

**Affiliations:** Wake Forest University School of Medicine, Winston-Salem, North Carolina, United States

## Abstract

This lecture will present the impact of cross disciplinary research mentoring and collaboration on improving outcomes for all of us as we age. Dr. Williamson will present the impact of the landmark concept article by Dr. Jim Fries, published in the New England Journal of Medicine (“Aging, Natural Death, and the Compression of Morbidity”) as a framework for his career. Among others, Dr. Williamson credits Dr. Linda Fried, his original mentor in this area, as he pursued a career focused on improving the health and well-being of older adults. The fruit of Drs. Fries’s work and Fried’s advice culminates in the work that he accomplished with his colleagues as part of the SPRINT and SPRINT MIND clinical trial. The lecture will encourage listeners to further advance the exploration of preventive gerontology as the best way to ensure that the care we provide is truly aligned with the needs of an aging population.

